# Living with constipation—older people's experiences and strategies with constipation before and during hospitalization

**DOI:** 10.3402/qhw.v11.30732

**Published:** 2016-04-26

**Authors:** Lene Munch, Nina Tvistholm, Ingelise Trosborg, Hanne Konradsen

**Affiliations:** 1 Institute of Nursing, Metropolitan University College, Copenhagen N, Denmark; 2 Pulmonary Department, Gentofte University Hospital, Hellerup, Denmark; 3 Research Unit, Gentofte University Hospital, Hellerup, Denmark

**Keywords:** Constipation, elderly people, lived experience, patient perspective, health-care professionals, strategies, interviews, qualitative research.

## Abstract

**Background:**

Constipation is a common problem among older people. This study aimed to explore how older patients experience constipation and which strategies they used in handling the condition before and during hospitalization.

**Methods:**

A qualitative exploratory research design was used. Fourteen semi-structured interviews were conducted with patients (61–91 years of age) during hospitalization. Data were analyzed by using content analysis.

**Results:**

Themes concerning experiences were *Bodily signs and symptoms of constipation*; the participants described severe pain during constipation, as well as pronounced relief after bowel movements, *Impact on well-being and social activities*; being constipated negatively impacted their mood and limited social activities, *Striving for bowel balance*; the participants experienced an ongoing strive for balancing between constipation and diarrhea. Themes related to strategies were *Struggling to find a solution*; they were aware of different strategies to prevent and treat constipation, though the most common solution described was the use of laxatives, *Wait and see*; the participants were awaiting to take action until they experienced constipation symptoms, *Constipation is a private problem being challenged during hospitalization*; constipation was considered a private issue rarely discussed with health-care professionals.

**Conclusion:**

This study illuminates the need for health-care professionals to be attentive to this issue and initiate the conversation with patients in order to advise on the management of constipation.

Constipation is a common problem, especially among older people (Spinzi et al., 2009). The prevalence varies in different studies (Higgins & Johanson, 2004), mainly due to differences in the definitions of constipation and study designs. The estimated prevalence of constipation among adults in the Western countries is approximately 15% (Wald et al., 2007), a number which increases with age (Spinzi et al., 2009). A recent systematic review estimated a prevalence of 33.5% among adults aged 60–101 (Mugie, Benninga, & Di, 2011).

There is no generally accepted international definition of the diagnosis of constipation. The existing definitions include symptoms like reduced frequency of defecation, hard and dry stool, and difficulties in defecation (Dal Molin et al., 2012; Spinzi et al., 2009). Yet, functional constipation is defined by the internationally acknowledged Rome III criteria (see Table I) as having ongoing symptoms for at least 3 months during the previous 6 months (Dal Molin et al., 2012). However, in contrast to the clear definition in the Rome III criteria, people tend to make their own individual definition of constipation based on a subjective assessment. One study found a discrepancy between how physicians and elderly people defined constipation, the physicians focused on a medical definition of a stool frequency of less than three times per week, while the primary symptoms for elder people were reduced frequency and having to strain in order to defecate (Whitehead, Drinkwater, Cheskin, Heller, & Schuster, 1989). Another study found that individual definitions on constipation were based on how the participants experienced their own bodies. The elderly decided for themselves in response to that experience, if they were constipated or not (Annells & Koch, 2002).

**Table I:** The Rome III criteria for functional constipation*

1. Fulfilling two or more of the following conditions:
(a) Straining during at least 25% of defecations
(b) Lumpy or hard stools in at least 25% of defecations
(c) Sensation of incomplete evacuation for at least 25% of defecations
(d) Sensation of anorectal obstruction/blockage for at least 25% of defecations
(e) Manual maneuvers to facilitate at least 25% of defecations (e.g., digital evacuation, support of the pelvic floor)
(f) Fewer than three defecations per week
2. Loose stools are rarely present without the use of laxatives
3. Insufficient criteria for irritable bowel syndrome

* Criteria fulfilled for the last 3 months with symptom onset at least 6 months prior to diagnosis.

ROME III, Appendix A: Diagnostic Criteria for Functional Gastrointestinal Disorders. (www.romecriteria.org/assets/pdf/19_RomeIII_apA_885-898.pdf)

Constipation is a condition caused by a variety of different physical, physiological, social, and pathological factors (Chatoor & Emmnauel, 2009; Richmond & Devlin, 2003). Constipation manifests itself in a range of physical symptoms, such as abdominal pain, straining, bloating, and nausea (Johanson & Kralstein, 2007). Apart from the physical symptoms, constipation seems to have a negative impact on other aspects of everyday life. Several studies have found that people with constipation generally have an impaired quality of life (Belsey, Greenfield, Candy, & Geraint, 2010; De Lillo & Rose, 2000; Norton, 2006; O'Keefe, Talley, Zinsmeister, & Jacobsen, 1995). In a population-based study among people suffering from symptoms of constipation, 73% of the respondents reported social or personal impairment related to constipation (Johanson & Kralstein, 2007). The decrease in health-related quality of life, observed in adults with constipation, has been found to be similar to the experience of people with chronic conditions such as osteoarthritis, rheumatoid arthritis, chronic allergies, and diabetes (Belsey et al., 2010).

Little is known about the experience of living with constipation in everyday life among elderly people. In a study, middle-aged women described constipation as a never-ending battle as they constantly had to adjust daily activities according to their bowel needs (Lämås, Anundsson, Stare, & Jacobsson, 2015). Another study among the elderly (above 65 years) found that participants were preoccupied with pursuing daily bowel movements, felt lethargic and tired, and that constipation had a negative impact on their mood (Mihaylov et al., 2008). Furthermore, constipation has been described as a cause of social isolation, (Koch & Hudson, 2000) and a group of frail elderly people described constipation as an intrusive condition, using words like terrible, awful, and horrible (Wolfsen, Barker, & Mitteness, 1993). Additionally, constipation is often described as a private issue (Lämås et al., 2015), and according to McClurg, Beattie, Lowe-Strong, and Hagen (2012), adults struggling with constipation are reluctant to talk about it with both relatives and health-care professionals.

Knowledge about elderly people's strategies to prevent and treat constipation is sparse. Most knowledge exists in relation to health-care professionals’ strategies (Gallagher & O'Mahony, 2009; Saga, Seim, Mörkved, Norton, & Vinsnes, 2014), and the few publications, exploring the strategies from the elderly patients’ perspective, focus on the use of laxatives (Annells & Koch, 2002; Mihaylov et al., 2008).

The symptoms of constipation tend to increase in numbers and severity during illness and hospital stays. A recent study identified symptoms of constipation in 44% of patients being admitted acutely to medical care unit. Furthermore, among those patients with no symptoms, 51% developed signs of constipation during the first 3 days of their hospital stay (Noiesen et al., 2014). Experiencing constipation during hospitalization tend to exacerbate the general well-being of the patients as well as prolonging the hospital stay (Harari, Martin, Buttery, O'Neill, & Hopper, 2007; Rasmussen & Pedersen, 2010; Trads & Pedersen, 2015). As a lot of patients already have or develop constipation during hospitalization, their usual strategies to manage and treat the condition might be difficult to initiate due to being ill and staying in other surroundings than home. Financially, constipation may lead to increased health-care costs due to factors such as increase in bed-days, use of laxatives, and extended use of nursing care (Sanchez & Bercik, 2011).

The prevalence of constipation among the elderly is increasing (Werth, Williams, & Pont, 2015), and the understanding of symptoms among patients with constipation needs to be expanded (Ervin et al., 2014). Clinical benefit of strategies used so far is unclear, and therefore, more attention toward patients’ experiences and strategies is needed (Spinzi et al., 2009). The strategies used at home to manage bowel habits need to be explored in depth; furthermore, these strategies might be under pressure during hospitalization, for example, due to patients’ reluctance to talk with health-care professionals about the issue (McClurg et al., 2012). Therefore, the aim of this study was to explore and identify how older patients experience living with constipation and which strategies they use in order to handle constipation before and during hospitalization.

## Method

This study used an exploratory research design, guided by qualitative content analysis.

### Participants

Patients admitted to a medical ward, in which patients with a broad variety of medical diagnoses were treated, participated in the study. Participants were purposefully sampled and recruited while still being admitted to hospital. A research assistant identified potential patients, and they were screened using the Rome III Criteria (see Table I). Inclusion criteria were being over the age of 60 years, meeting criteria for constipation according to Rome III, living in their own home before being admitted as well as being able to communicate in Danish. Patients with bowel cancer or other gastrointestinal diseases were excluded. The age limit of 60 years was chosen, as this age seems to indicate an increase in the prevalence of constipation (Mugie et al., 2011).

Fourteen patients were included in the study, seven males and seven females with ages ranging from 61 to 91 years. At the time of the interview, the participants had been hospitalized for durations ranging from 1 day to 5 weeks.

### Data collection

Data were collected from September 2014 to January 2015.

Patients who agreed to participate were interviewed in private surroundings while they were still in hospital. The researcher repeated the information on the study and once again obtained informed consent from the patients verbally and in writing.

The interviews were face-to-face and carried out by trained interviewers. A semi-structured interview guide was used. Questions were developed from the existing research literature. The central questions were open-ended and revolved around: the experience of being constipated at home and during hospitalization, and the impact constipation had on everyday life as well as the routines, measures, and strategies used to prevent and treat constipation. The average duration of interviews was 17 min—ranging from 6 to 45 min. The interviews were tape-recorded and transcribed verbatim by a research assistant, and transcripts were reviewed for accuracy by the interviewers. Being constipated is often discussed as a private issue (Friedrichsen & Erichsen, 2004; Lämås et al., 2015; McClurg et al., 2012) and confidence became an important part of the atmosphere during the interviews. The interviewers were attentive to how each participant managed the interview situation.

### Ethical approval

The participants were reassured that participation was entirely voluntary and that they could withdraw from the study at any time. They were guaranteed confidentiality in the presentation of the findings. The study was evaluated by the National Committee on Health Research Ethics (number H-4-2014-FSP) and approved by the Danish Data Protection Agency (number 2014-41-3091).

### Data analysis

Interviews were transcribed shortly after having taken place. Transcribes were then analyzed using qualitative conventional content analysis (Hsieh & Shannon, 2005). The goal was to derive meaning from the interviews and to identify recurring conceptual patterns of experience across the data material. No predefined theory was used to explore the participants’ experiences, which is recommended when existing knowledge about the issue is sparse and the analysis aims to move from a specific into a general level of knowledge (Graneheim & Lundman, 2004).

Analysis took place in phases in which all authors participated. At the first step, the authors listened through the audio recordings and carefully read and reread transcriptions to become familiar with the content of each interview. Next step was the identification of meaning units by collecting words or statements relating to the same central meaning. Each meaning was labeled with a code. This part was performed as a dialog between the authors. The meaning units were condensed and sorted into categories related to their content. A category refers to a descriptive level of content. The interpretation process resulted in themes, which are threads of meaning that run through meaning units of codes on an interpretative level (Graneheim & Lundman, 2004).

In order to enhance validity and trustworthiness, recommendations for inductive analysis were used (Elo & Kyngas, 2008). This meant that when inconsistencies occurred in the coding or when making themes, decisions were justified on the basis of data and the researchers found consensus in the course of discussion.

## Results

In this study, participants reported that being constipated meant a pervasive experience and they explained many strategies to handle the condition. The themes *bodily signs and symptoms of constipation, impact on well-being and social activities*, and *striving for bowel balance* illustrated experiences; and the themes *struggling to find a solution*, *wait and see*, and *constipation is a private problem being challenged during hospitalization* were related to the patient's strategies.

Being constipated was associated with subjective, personal, and comprehensive experiences, which had a negative impact on everyday life and well-being before and during hospitalization. The participants described several effects constipation had caused on both their bodily, mental and social well-being.

### Bodily signs and symptoms of constipation

A variety of bodily symptoms were associated with being constipated, especially abdominal pain. Pain was described as either being a kind of pressure or a severe pain. One patient said: “(it) has been such a pressing pain” (P14), while another participant described his painful abdomen as “I felt that it was about to burst” (P1). Other bodily symptoms described, were feeling bloated, uncomfortable and tired, as described by a participant: “Yeah, the stomach is almost growing….and gets heavy” (P12), and finally summing it up as very bothering.

Metaphors were used to describe the bodily experience of constipation and the main themes of these metaphors were allocated to either the throat or the end of the intestine. Some patients had a feeling of “having it up to the throat” (P5), while others were feeling blockage at the end of the intestine. One participant explained that: “one cannot get rid of it. It is just as if there was a plug down there” (P4).

During the periods of constipation, the participants often experienced “false alarms,” which were associated with a feeling of being disappointed. They felt the pressure of a bowel movement, but most often there was no success when they acted on the signal and went to the toilet. It was described by one participant: “As soon as I can sense a little bit uhh now it is there, I hurry out…and then it is just gas” (P12). Another said: “I think I have to go to the toilet, and then I go out there and press and press and press, and then nothing come any way…Well at least no more than a small, rock hard lump” (P13). Defecating was painful and often resulted in stinging and bleeding due to rectal lacerations and was compared to “giving birth” (P3). An older man described his latest visit to the toilet: “I see lighting in front of my eyes, I was about to fall off the toilet” (P1).

The only relief to the discomfort of constipation symptoms was having a bowel movement. This was described by one respondent as a feeling of “being newborn, when you have gotten rid of such an amount” (P3). Constipation and defecation seemed to challenge the participants’ strength, and several described how exhausted they ended up being “I push like crazy…afterwards I had to go and lie down, I slept for two hours” (P1).

### Impact on well-being and social activities

Frustration in relation to the inadequate functioning of the gastrointestinal system was experienced in different ways. Constipation impacted the mood and general well-being, with feelings of discomfort, worry, and even a kind of desperation. Not having a stool could spoil the day, as it was leading to a certain degree of mental pressure and stress. A broad spectrum of feelings were activated and maintained by the presence of constipation. One of the participants described his mood while constipated: “I will tell you in plain Danish, it is bloody depressing” (P1).

During the periods of constipation, quality of life was affected and followed by feelings of spoiled optimism, bad mood, and energy deficits. The fatigue and exhaustion seemed to be reasons to avoid and miss social activities, being tired depleted the remaining energy to go out and meet other people. Some also expressed the need to stay close to a toilet all the time. In order to feel relaxed, some had to know exactly where to go, if something started which limited social activities. But it was not only the constipation causing these limitations, problems related to the prevention and treatment of the condition could also keep the informants at home. One explained: “Then when I have diarrhea, it is like hell …. I almost have to go and stare at the closest toilet” and he continued: “we have installed at toilet in the car, just a precaution” (P1).

### Striving for bowel balance

Overall, the participants were striving to balance their bowel habits. They seemed to suffer from unregulated bowel habits, as constipation could easily switch to diarrhea. Either the stool was too soft or too hard. It was a continuous effort to achieve balance, but it seemed that the goal was hard to reach. As explained by one participant: “I don't know really. It is like…then it changes to the opposite and becomes thin, then I have to go the opposite way to stop it” (P1). Another one said: “And then it was loose stools, and then it was too thick. Yeah then I could not get rid of it” (P5). Not reaching the toilet in time was especially embarrassing, one participant told of an incident during his stay in hospital when he had taken too much laxative: “We almost soiled the whole hotel (hospital), the whole living room – every step, it really was pure shit, so to speak” (P3).

### Struggling to find a solution

Patients often tried to find plausible explanations for why the symptoms of constipation frequently arose. The explanations were grounded in personal experiences and often not based on discussions with health-care professionals “I think I am born with a dysfunctional bowel, even though my doctor says it's not true” (P14). Some participants wondered about whether constipation was self-inflicted “yes then I think – have you eaten something that has brought you in this situation” (P11) and sometimes even avoiding certain food items “For my part, I do not eat cheese or drink milk and I only eat organic flour” (P11).

The most frequently used solution to constipation was laxatives. Lifestyle adjustments were thought worthy of consideration but were not necessarily viewed as a solution. The participants felt that they had tried exercise, different foods, and drinking more fluids or that their physical or economic situation stopped them from changing these factors. As one man said, “I used to climb the stairs at least four times a day. But then the stairs got too difficult to manage for my wife, and we moved to the ground floor. I stopped getting that exercise, and now I don't think I can anymore” (P5).

Even though laxatives were believed to be the solution, it was also thought that the balance between constipation and diarrhea was impossible to manage: “It can change from one minute to the next” (P3). Which medication to take was often a difficult decision: “There is so much advice; it is hard to find the balance of how much medicine one should take” (P3). Experience of taking different kinds of laxatives did not necessarily help this decision: “The thing you dissolve in water, it helps but you have to be very careful not to take too much or too little” (P7). One strategy might, therefore, be to get it over with, and then again wait and see how it would turn out the next time, as one woman said: “Sometimes I give it an extra shot, and then everything comes out and I can wait for the next time it stops” (P1). The strategies used were all related to accepting constipation as a condition that comes and goes.

### Wait and see

While at home the strategy was to wait and see, and this strategy was continued when hospitalized. “I wait three to four days, and then if nothing happens, then I know I have to do something” (P5). The patients often experienced constipation for many years, either having lived with the symptoms being present now, and for some, all their lives or since coming of age. The strategy of wait and see was often grounded in the experience of constipation being a condition which was very difficult to control. According to this man, who was spending his fourth day at the hospital without being to the toilet: “When I get home I will give it (the stomach) some extra. At home I am in control of what medicine to take” (P14). Patients waited to talk about their bowel movements, believing that if it was important the nurses would raise the issue “No, I have not asked the nurse for advice, they know very well what my problem is. If there is anything else I need to know I expect they will come to me” (P14).

### Constipation is a private problem being challenged during hospitalization

Some participants described using the hospital toilet as an experience of insecurity and stress due to the fact that the hospital was not their home. It was quite a challenge for patients to transfer toilet habits from home to the hospital setting. One of the participants expressed a feeling of more peace and quiet at home and explained: “…of course it is different knowing we are so many sharing one toilet – you cannot allow yourself to sit there for a quarter of an hour, because you imagine a line of older ladies and gentlemen, standing outside the door, freezing” (P14). Several of the participants said that they did not feel relaxed when using the hospital toilets.

Being admitted meant expecting the constipation to worsen, as the usual habits of exercising, eating, and drinking were difficult because of the ailment that was the reason for the hospitalization. Expecting constipation to either appear again or worsen was not discussed with health-care professionals at the hospital. “It is a delicate subject” (P6), one woman said, and “It is not the reason I am here” (P6). A man who needed help to wash himself after being to the toilet and who was used to his wife helping him at home, had never asked for help at the hospital. Instead, the problems were solved by discussing the issue with close relatives or planning to solve it when getting home. Another man said: “I have talked to my wife about it and she knew the name of a laxative which I will buy at the pharmacy. I have not talked to the doctors and nurses about it, or mentioned that I take these laxatives at home. I have noticed that they do not like that you self-medicate” (P10). None of the patients would hesitate to mention it if they wanted to, but for these participants, constipation was considered to be a personal problem, and they felt that it was up to them to deal with it.

## Discussion

In this study of older persons’ experiences and strategies related to constipation before and during hospitalization, a picture of a painful and private condition that was hard to manage was illuminated.

Numerous studies have described the discomfort, pain, and decreased quality of life related to constipation (Miles, Fellowes, & Wilkinson, 2009; Spinzi et al., 2009; Wald et al., 2007), which were also identified in the present study. This study offers an additional insight into the strategies used, which might often be hidden, as patients described them as being personal and not discussed with health professionals.

Striving for bowel balance and struggling to find a balance might be related to constipation being a condition for which many try to self-medicate (Bliss, Fischer, & Savik, 2005; Tack et al., 2011). It has been shown that in relation to fecal incontinence patients found the best treatment by trying different options by themselves before talking to health-care professionals and mainly needed professionals to provide hope for improvement (Cichowski, Dunivan, Rogers, & Komesu, 2014). This might also be the case in relation to constipation among the elderly, as these are patients with prior, and sometimes very long, experience of the condition.

Being a private challenge and personal responsibility, self-medication might on the one hand empower patients and let them play an active role in finding the best solution for treating constipation. On the other hand, laxatives may interact with some (but not the most common) drugs and result in incorrect use of medication (Ruiz, 2010; Singh, Chaudhary, Azizi, & Green, 2014) and sufferers becoming over- or under-medicated or even masking symptoms of severe diseases. The symptoms of constipation might also be difficult to interpret, as the association between the use of laxatives and self-reported constipation is poor (Werth et al., 2015).

The strategies that the patients elucidated in this study are strategies that are not necessarily clinically effective in relation to solving the condition of being constipated. Using the strategy of wait and see and considering constipation a private challenge may also be related to not having discussed this issue during hospitalization. Other studies have found that prevalence and incidence are high, and often health-care professionals do not act until the problems are severe (Cardin et al., 2010; Noiesen et al., 2014; Rasmussen & Pedersen, 2010). On the one hand, this may be due to the fact that constipation remains difficult to talk about for both the patient and the nurse, like other bowel symptoms (Bliss et al., 2005; Cichowski et al., 2014) or other issues such as sexuality (Farrell & Belza, 2012) and spirituality (Molzahn & Sheilds, 2008). Silence might also for patients serve as a protection against feeling to have to talk about an embarrassing issue, as it has been found among patients with fecal or urinary incontinence (Bliss et al., 2005; Higa, Chvatal, De Moraes Lopes, & Turato, 2011). On the other hand, the evidence from non-pharmacological and pharmacological treatment of constipation in elderly patients is unclear, so nurses might be unsure what to recommend (Muller-Lissner, Kamm, Scarpignato, & Wald, 2005; Rao & Go, 2010; Spinzi et al., 2009). Research has suggested that increased recording of stool frequency, counseling on bowel training, fiber and fluid intake, and mobilization (Hsieh, 2005) may treat constipation in older adults, but also that there are numerous suggestions for interventions and that they likely have to be individual (Bouras & Tangalos, 2009). The strategy of wait and see might therefore be helpful when trying out different suggestions for managing constipation, as the best way forward is to rely on one's own experiences (Bosshard, Dreher, Schnegg, & Bula, 2004), as the evidence on how to treat constipation in old age is insubstantial (Gallagher & O'Mahony, 2009).

However, studies of constipation management among older adults in primary care have shown that prevalence of constipation is high before hospitalization (Noiesen et al., 2014; Song, 2012) and that it is one of the symptoms that, among older adults, adds to a symptom's burden which is associated with higher risk of hospitalization (Salanitro et al., 2012). Then when hospitalized they meet nurses who prioritize routine tasks, which may contribute to the risk of overlooking the issue and neglecting individual strategies both in the prevention and treatment of constipation (Saga et al., 2014). More research is needed to understand what strategies and specific interventions nurses consider and how they choose among these, when they meet older adults with signs of constipation both in the home care setting and at the hospital.

The impact of constipation resulted in decreased social activity, which might mean that, for periods, days are planned around bowel movements (Bouras & Tangalos, 2009). Others have suggested that decreased social activity might be a result of constipation being painful, an unstable condition and of being difficult to talk about (Friedrichsen & Erichsen, 2004; Lee & Warden, 2011). The predictability and regularity of defecating has been found to be perceived as important among older adults (Mihaylov et al., 2008).

Patients who were included in this study might have had different underlying diseases causing constipation, even though not diagnosed. Whether this has influenced the experiences of the patients is unknown. This is also the case related to the exploration of the patients’ strategies, as undiagnosed diseases might be the reason for why some strategies might not work. Though, all participants fulfilled the Rome III criteria meaning that they have had ongoing constipation symptoms for at least 3 months during the previous 6 months. The interviews were of different length. A few of them were short due to some patients being very tired at the time of the interview. Furthermore, one interview was stopped after a few minutes, as the patient suddenly felt unwell. Another possibility is that for some people, the subject might have been difficult to talk about. On the other hand, important knowledge came from the interviews.

Future studies are needed in order to expand our knowledge around the possible interplay between experiences and strategies, and whether certain experiences lead to certain strategies.

This study explored patients’ experiences before and during hospitalization; following the patients after hospitalization might have expanded the knowledge which it presents. Having interviewed the patients in private surroundings and having recovered from the illness that brought them to the hospital might also have influenced the dialog between interviewer and interviewee especially as constipation is an issue that, for some patients, is a delicate subject.

## Conclusions

Constipation is common, unpleasant, and interferes with older peoples’ overall well-being. It is an ongoing battle for many to balance their lives around toilet visits, before, during, and possibly after hospitalization. As neither patients nor health-care providers bring up this topic during hospitalization, health-care professionals must be aware of and initiate conversations regarding this issue. The professionals need to listen to the strategies patients have in order to advise on how to manage symptoms and prevent further constipation-related decline during hospitalization.
